# Tech-Tuned Smiles: A Narrative Review on the Role of Software in Modern Orthodontics

**DOI:** 10.7759/cureus.92706

**Published:** 2025-09-19

**Authors:** Akshay Puthenpurayil, Rahul D Prabha, Sapna Varma N K, Ajith Vallikat Velath

**Affiliations:** 1 Orthodontics and Dentofacial Orthopedics, Amrita School of Dentistry, Amrita Institute of Medical Sciences, Kochi, IND

**Keywords:** artificial intelligence (ai), automatic planning, digital dentistry, digital orthodontics, digital software

## Abstract

Digital orthodontic treatment has revolutionized modern orthodontics by integrating advanced software solutions into diagnosis, treatment planning, and appliance fabrication. These software systems optimize clinical workflows, increase accuracy, and enhance patient outcomes. These include 3D imaging tools for capturing intraoral scans, cephalometric analysis for precise measurements, and computer-aided design/computer-aided manufacturing (CAD/CAM) technologies for custom orthodontic appliances and software for predicting tooth movement and customizing aligner sequences, allowing virtual treatment simulations to visualize outcomes, and boosting confidence and compliance. Intraoral scanners capture digital models quickly, replacing traditional impressions, saving time and improving comfort. Moreover, automated patient management tools track progress and send reminders, keeping treatments on schedule. Despite these advantages, the high costs of software, the learning curve for practitioners, and the need for constant updates can be challenging. This abstract provides an overview of digital orthodontic software's pivotal role in advancing personalized, efficient, and precise orthodontic care.

## Introduction and background

**Figure 1:**
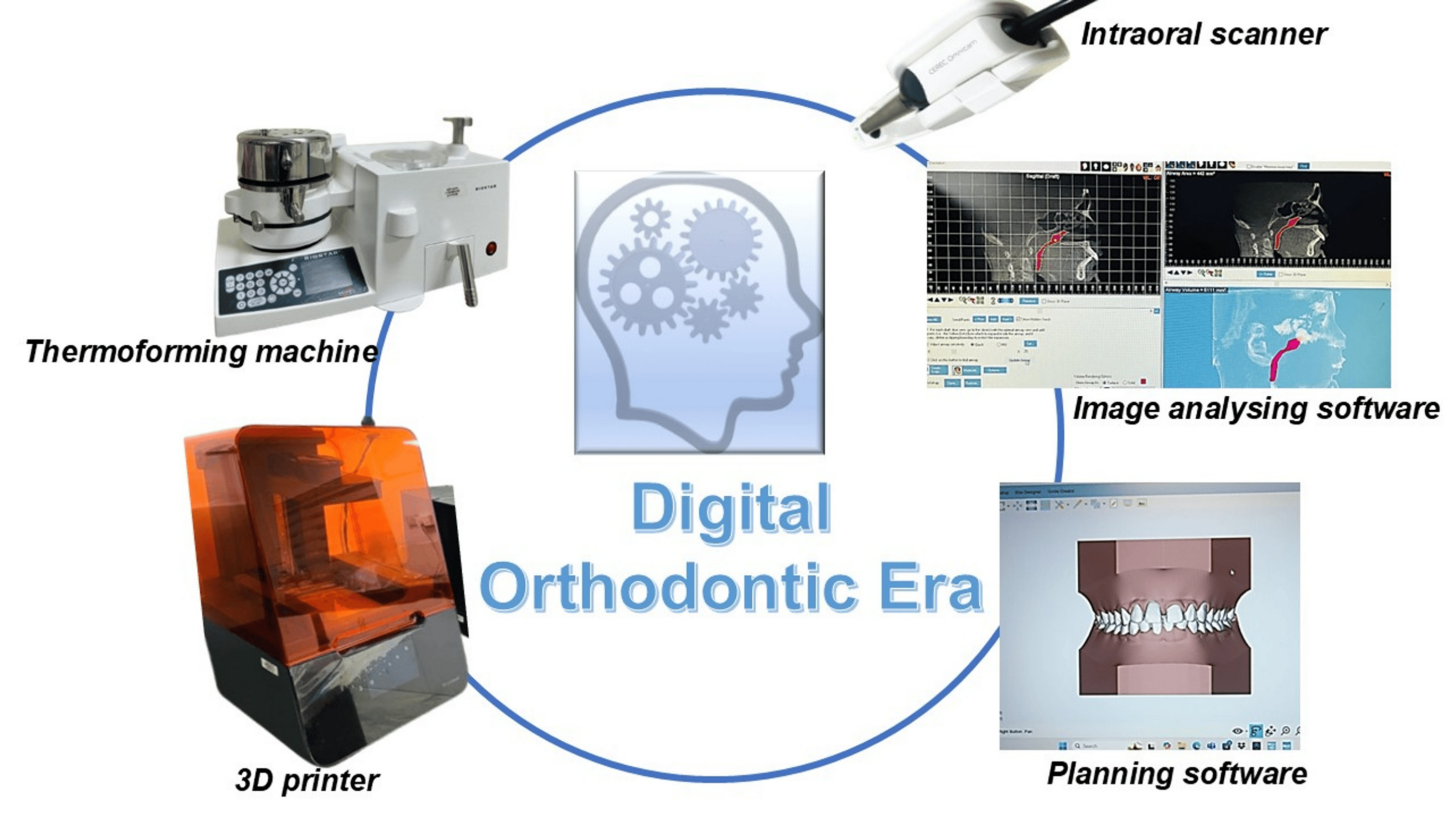
Workflow of digital orthodontics

Digital dentistry and artificial intelligence (AI) advancements have significantly improved orthodontic diagnosis, treatment planning, and outcome predictions (Fig. 1). AI provides valuable support by delivering rapid, precise insights while minimizing human error. However, it does not replace the orthodontist’s expertise; clinicians still make the final call on diagnoses and treatment plans. Modern innovations, like cone beam computed tomography scans, 3D imaging, intraoral/facial scanners, dental modeling software, and 3D printing, are reshaping orthodontic care. These tools enhance anatomical understanding and enable personalized, dynamic 3D treatment planning, contributing to efficient and effective orthodontic treatments, benefiting clinicians and patients [1]. Deep learning (DL), a branch of AI based on multilayered neural networks, further strengthens orthodontic applications. Models such as convolutional neural networks (CNNs) for image analysis, recurrent neural networks (RNNs) for sequential data, generative adversarial networks (GANs) for outcome simulation, and autoencoders for image reconstruction are increasingly used in cephalometric analysis, airway segmentation, growth prediction, and remote treatment monitoring. Their integration into clinical workflows marks a paradigm shift toward more precise and individualized orthodontic care. This comprehensive review outlines the clinical applications (Table 1) of orthodontic software used for diagnosis and treatment planning.

**Table 1:** Overview of software used in orthodontics

Clinical applications	Software
Cephalometric analysis	WebCeph (AssembleCircle Corp., S. Korea)
	Dolphin (Patterson Dental Holdings, USA)
	Nemoceph (Nemotech, Spain)
	VistaDent (Dentsply Sirona, USA)
	CephNinja (Cyncronus LLC, USA)
	OneCeph (NXS, India)
	FACAD (Ilexis AB, Sweden)
	OrisCeph (Elite computer Italia, Italy)
	OnyxCeph (Image Instruments GmbH, Germany)
Patient management	Dolphin (Patterson Dental Holdings, USA)
	Aquarium (Patterson Dental Holdings, USA)
	Dental Monitoring (Dental Monitoring SAS, France)
	Open Dental (Open Dental Software, Inc., USA)
	DentOne (DIORCO, Korea)
	Titan (Titan Dental Design, USA)
Model analysis	OrthoAnalyzer (3Shape, Denmark)
	DigiModel (HyTech, Inc., USA)
	RapidForm (3D Systems, North Carolina)
	OrthoCAD (Align Technology, USA)
	eModel (GeoDigm Corp., USA)
	Anatomodel (Anatomage, USA)
	ivoris® analyze (Computer konkret AG, Germany)
	MeshLab (Visual Computing Lab, Italy)
	03D (Widialabs,Brazil)
	Meshmixer (Autodesk Inc., California)
Aligner	ClinCheck Pro 6 (Align Technology, USA)
	Maestro 3D (AGE Solutions, Italy)
	OrthoAnalyzer (3Shape, Denmark)
	Deltaface (Coruo, France)
	OnyxCeph (Image Instruments GmbH, Germany)
	SureSmile (Dentsply Sirona, USA)
	Blue Sky Plan (Bluesky Bio, USA)
	Direct Aligner Designer (DAD) (Graphy, S.Korea)
Smile analysis and smile designing	SureSmile (Dentsply Sirona, USA)
	Smile Analyzer (Mashhad Faculty of Dentistry, Iran)
	Digital Smile Design (DSD, Brazil)
	ImageJ (National Institutes of Health, USA)
	Exocad (Align Technology, USA)
	Adobe Photoshop (Adobe Inc., USA)
	3Shape Smile Design (3Shape, Denmark)
	ClinCheck (Align Technology, USA)
Splint and orthognathic surgical planning	Meshmixer (Autodesk Inc., California)
	Maestro 3D (AGE Solutions, Italy)
	Dolphin (Patterson Dental Holdings, USA)
	Materialise Mimics (Materialise, Belgium)
Treatment planning	SureSmile (Dentsply Sirona, USA)
	Digital Smile Design (DSD, Brazil)
	Dentone (DIORCO, Korea)
Indirect bonding	Orthoanalyzer (3Shape, Denmark)
	SureSmile (Dentsply Sirona, USA)
	Maestro 3D (AGE Solutions, Italy)
Metal 3D printing	Exocad (Align Technology, USA)
	Meshmixer (Autodesk Inc., California)
	Blue Sky Plan (Bluesky Bio, USA)
	3Shape (3Shape, Denmark)
	Blender (Blender Foundation, Netherlands)
	Materialise Magics Module (Materialise, Belgium)
Face scan and 3D planning	Planmexa Romexis (Planmeca Oy, Finland)
	DentSim (Image Navigation, Israel)
	CEREC (Dentsply Sirona, USA)
	Exocad (Align Technology, USA)
	OrthoCAD (Align Technology, USA)
	Kodak 3D Imaging (Carestream Dental, USA)
	Meshmixer (Autodesk Inc., California)
	Meshlab (Visual Computing Lab, Italy)
	Titan (Titan Dental Design, USA)
	Blender (The Blender Foundation, Netherlands)

## Review

Methodology

Search Strategy

For this narrative review, a comprehensive literature search was conducted across major electronic databases, including PubMed/MEDLINE, Scopus, Web of Science, Embase, and Google Scholar. Additional manual searches were performed in reference lists of included articles and relevant orthodontic/dental journals to ensure completeness. The search included publications from 2000 to 2025 to capture both foundational and recent advancements. The following keywords and Medical Subject Headings (MeSH) were combined using Boolean operators: (“Orthodontics” OR “Dentofacial orthopedics”) AND (“Digital dentistry” OR “Artificial intelligence” OR “3D imaging” OR “Cone beam computed tomography” OR “Cephalometric analysis” OR “Dental software” OR “Aligner therapy” OR “Digital smile design” OR “3D printing” OR “Virtual surgical planning”).

Inclusion Criteria

Articles written in the English language and published between 2000 and 2025 were included. Eligible study designs included randomized controlled trials, cohort studies, case-control studies, cross-sectional studies, systematic reviews, scoping reviews, narrative reviews, and clinically relevant case series. Priority was given to research on AI, imaging software, cone-beam computed tomography, cephalometric analysis platforms, virtual treatment planning, aligner therapy, digital smile design, 3D printing, or computer-assisted surgical workflows in orthodontics. 

Exclusion Criteria

Studies were excluded if they were not published in English, involved animal or in-vitro experiments, or consisted of purely technical or engineering reports without direct orthodontic application. Editorials, letters to the editor, commentaries, and conference abstracts lacking sufficient methodological detail were also excluded. Articles unrelated to orthodontics, such as those limited to prosthodontics or implant-only applications, were not considered. In addition, duplicate records and studies with incomplete or inaccessible full texts were excluded from the review.

Cephalometric analysis

Manual cephalometric landmark identification can be tedious in busy practices, with results varying based on X-ray quality and the clinician's judgment. Advancements in fully automated cephalometric landmark identification have significantly enhanced clinical efficiency and its potential use in routine clinical applications. AI has proven to be a time-saving and dependable tool, facilitating routine cephalometric tracing for diagnosis, treatment planning, and generating multiple datasets for research [2]. The cloud-based, AI-driven WebCeph platform (AssembleCircle Corp., South Korea) is notable for its automated cephalometric analysis and superimposition, fast processing, cross-platform interoperability, and installation-free cloud storage [3]. By contrast, Dolphin Imaging (Patterson Dental Holdings, USA) demonstrates digital analysis process with accuracy for cephalometric analysis and treatment planning [4]. Nemoceph 13 (Nemotech, Spain) is a software known for its strong diagnostic capabilities, including growth prediction and soft tissue simulations, as well as its ability to blend photos and X-rays, making it suitable for case presentations [5]. VistaDent OC (Dentsply Sirona, USA) presents reliable options for 2D and 3D measurements and excels in simulating both bone and soft tissue changes [6]. CephNinja (Cyncronus LLC, USA) provides a portable, AI-powered solution for cephalometric analysis, offering an alternative to traditional software, with an emphasis on quick results and user-friendliness, particularly on mobile devices [5]. OneCeph (NXS, India) also offers a mobile solution with automatic measurements and easy export options, making it ideal for chair-side use [7]. FACAD (Ilexis AB, Sweden) provides a versatile tool for both digital tracing and soft tissue prediction, supporting over 28 predefined analyses and offering customization, making it a reliable option [8]. OrisCeph Rx3 (Elite computer Italia, Italy) brings unique features like superimposition, morphing, and Virtual Treatment Objectives (VTO), allowing for tailored treatment planning and custom charting [9]. Lastly, OnyxCeph (Image Instruments GmbH, Germany) stands out for its speed and accuracy in digital cephalometric analysis but may require manual adjustments for soft tissue profile predictions, with the added benefit of integration with OnyxDBServer for multi-facility use [10].

Patient management

Dolphin Patient Management software (Patterson Dental Holdings, USA) integrates seamlessly with digital imaging tools to improve orthodontic diagnostics and treatment planning, offering precise predictions of tissue changes in orthodontic treatment, orthognathic cases and aids in comprehensive treatment planning in complex cases [11]. In comparison, the Dolphin Aquarium (Patterson Dental Holdings, USA) uses 3D graphics to visually demonstrate orthodontic procedures, appliance use, and treatment options.

The spectrum of orthodontic patients has widened with clear aligner therapy. The remote treatment progress monitoring is facilitated by treatment monitoring software. The relevance and usage of remote treatment monitoring using software has increased post-pandemic [12]. Dental Monitoring (DM) (Dental Monitoring SAS, France) is an integrated system comprising a mobile app for patients, a web-based Doctor Dashboard®, and a movement-tracking algorithm that analyzes patient-submitted photos. DM aims to reduce in-office visits, detect aligner issues and misfits, and personalize treatment [13]. Studies showed benefits of DM in orthodontic care, including reduced chairside time, improved patient compliance, early detection of emergencies, reduced relapse, remote aligner fit monitoring, and better oral hygiene management [13,14]. Open Dental (Open Dental software, Inc., USA) is a versatile, all-in-one practice management software that supports scheduling, charting, billing, and patient records and also facilitates tele-dentistry, allowing remote consultations and diagnostics, which has become increasingly important for modern practices [11]. Apart from automated cephalometric analysis, including landmark identification, WebCeph (AssembleCircle Corp, South Korea) has a patient management platform that offers a comprehensive suite to streamline orthodontic workflows, improve patient education, and enable precise treatment planning and record maintenance [3]. DentOne (DIORCO, Korea) is a software tailored to support orthodontic practices in managing patient data, appointments, treatment plans, and finances. By automating key processes it helps reduce administrative burden, improve patient communication, and enhance the overall efficiency of the practice [15]. Titan (Titan Dental Design, USA) is a comprehensive tool for 3D imaging, treatment planning, patient management, and automated administrative functions that reduce reliance on multiple software tools, making it a valuable choice for modern orthodontic practices [13].

Model analysis

Digital models have widely replaced plaster casts, providing more accurate measurements and efficient assessments using specialized software.

OrthoAnalyzer (3Shape, Denmark) is a powerful tool for creating databases, mapping bite colors, conducting 2D cross-sectional and Bolton analyses, and measuring tooth widths, making it superior to traditional manual methods. Similarly, DigiModel (HyTech, Inc., USA) automates orthodontic model analysis, Bolton analysis, and tooth width measurements, providing accuracy and clinical validity, thus enhancing orthodontic assessments [16]. Rapidform (3D systems, North Carolina) stands out for its 3D modeling capabilities, integrating CBCT data to analyze tooth movements and occlusal contact areas, offering precise pre- and post-treatment scan superimposition [17]. OrthoCAD (Align Technology, USA) produces 3D digital models from physical impressions, proving its effectiveness in Bolton analysis and arc length deviation, and integrating well with intraoral scanners like iTero Element 2 and CBCT-generated models [16]. From the 3D modeling perspective, eModel (GeoDigm Corp., USA) uses laser scanning for bite capture and includes tools like collision detection and a digital articulator, offering diagnostic accuracy and faster processing than traditional methods [18]. Anatomodel (Anatomage, California, USA) is a 3D digital study model created directly from cone beam computed tomography (CBCT) scan data, eliminating the need for traditional impressions. This advancement minimizes patient discomfort and saves orthodontists valuable time, reducing the need for physical impressions, staff involvement, and materials [19]. Ivoris Analyze3D (Computer konkret AG, Germany) produces digital models with slightly larger tooth size measurements but offers more consistent results than manual analysis, showcasing the benefits of a digital workflow [20]. MeshLab (Visual Computing Lab, Italy), an open-source software, processes 3D meshes for digitization and printing, with features like alignment and model comparison, making it reliable for evaluating 3D printed models [21]. O3D (Widialabs, Brazil) allows orthodontists to access, download, and analyze digitized dental casts. It enables precise measurements of parameters like dental arch size, arch width, overjet, and overbite [22]. Finally, the versatile Meshmixer software (Autodesk Inc., California) presents as an affordable tool for mesh repair, STL file editing, and occlusion simulation, making it useful for orthodontic diagnostics and presurgical modeling [23].

Aligner therapy

Several software platforms are available in the market for aligner therapy that assist orthodontists in treatment planning, simulation, and monitoring the progression of aligner treatments.

ClinCheck Pro 6 (Align Technology, USA) is a proprietary cloud-based tool for 3D treatment planning that supports features like attachment placement, power ridge, and interproximal reduction [24]. Maestro 3D (AGE Solutions, Italy) stands out with its capability for 3D modeling, tooth repositioning, in-house aligner setup and appliance design, and export models for 3D printing. It delivers highly accurate measurements and customizable models, with 3D-printed aligners outperforming traditional thermoformed ones in precision [25]. OrthoAnalzer (3Shape, Denmark) enables precise analysis of 3D digital scans and CBCT data, aiding in the creation of detailed treatment plans with features like tooth movement simulation, root position visualization, and a comprehensive toolset for designing aligners and appliances [25]. Deltaface (Coruo, France) also specializes in clear aligner design, offering advanced features like CBCT data integration for enhanced tooth adjustments and aligner customization, making it a strong tool for orthodontists aiming to refine mechanical properties and patient outcomes. It allows the thickening of the aligner in specific areas, where the operator wants to have a stiffer part [26]. OnyxCeph3™ (Image Instruments GmbH, Germany) supports 3D space calculations, virtual treatment planning, and aligner design, frequently used in research on maxillary expansion and aligner force transmission with 3D printed models. Its comprehensive project-saving capabilities and treatment design tools are beneficial for complex cases [27]. SureSmile (Dentsply Sirona, USA) leverages 3D impressions to create custom aligners, improving treatment speed and results, with outcomes dependent on the quality of its virtual setup [24]. Blue Sky Plan (BSP) (Bluesky Bio, USA) excels in converting CT or CBCT scans into 3D models for precise orthodontic and implant planning, facilitating aligner production, tooth movement, and 3D printing [28]. Direct Aligner Designer (DAD) (Graphy, South Korea) is an advanced tool that automates treatment planning, tooth movement simulations, and aligner designs, boosting precision and efficiency while reducing manual work [29]. Unlike tools like ClinCheck Pro, ArchForm, or SureSmile, DAD emphasizes quicker treatment planning and enhanced customization through AI, leading to better clinical outcomes and patient satisfaction.

Smile analysis and smile designing

In orthodontics, smile analysis combines aesthetic goals and physical constraints to create personalized treatment plans, using digital tools to assess lip-tooth dynamics. Each software brings unique features to improve results and patient satisfaction, advancing digital smile design in orthodontics.

ClinCheck In-Face Visualization Tool (Align Technology, USA) offers facial rendering for real-time treatment planning, multi-layer visualization, restorative 3D modifications, and tooth mass analysis, helping patients visualize results and enhancing personalized orthodontic care [30]. SureSmile (Dentsply Sirona, USA) is a 3D imaging-based technology that customizes archwires with robotic precision, reducing treatment time by up to 34% and enhancing accuracy. Its key features include custom wire sequencing, optimized planning tools, indirect bonding, and bracket placement. The integration of cloud technology ensures secure patient data storage, making it a comprehensive system for efficient treatment planning and execution [31]. Smile Analyzer (Mashhad Faculty of Dentistry, Iran) is a software tool designed for analyzing lip-tooth relationships and facial landmarks during various facial expressions. It includes a set of measurement tools for point and line placement on images, which helps orthodontists and cosmetic dentists assess smile aesthetics effectively [32]. Digital Smile Design (DSD, Brazil) developed by Christian Coachman in 2007, takes smile design to the next level by combining facial, gingival, and intraoral analysis, followed by digital adjustments to the teeth’s size and position. It is a strong tool for customized smile makeovers since it is focused on patient communication and provides real-time feedback with digital mock-ups. DSD uses cameras, intraoral scanners, and 3D printers to refine and visualize the treatment [33,34]. ImageJ (National Institutes of Health, USA) is a free, open-source image processing software that measures distances, angles, and proportions in 2D and 3D images. It helps with smile design analysis, bone level measurements, tooth morphology, and assessing gingival pigmentation [35]. Exocad (Align Technology, USA) is a dental CAD software for smile design and restorations, featuring TruSmile for photorealistic rendering and Smile Creator for 2D and 3D design. It is useful for TMJ treatment, smile makeovers, and crown lengthening. Finally, Adobe Photoshop (Adobe Inc., USA), traditionally known for image editing, also plays a role in smile design through the Photoshop Smile Design (PSD) technique. It allows clinicians to digitally adjust tooth dimensions on patient images, aiding in treatment planning and enhancing communication between patients and ceramists. 3Shape Smile Design (3Shape, Denmark) uses photographs and optical impressions with the RealView Engine for realistic prosthesis design, integrating seamlessly with 3Shape CAD/CAM systems [34].

Splint and orthognathic surgical planning

Software platforms are available and specifically designed for splint design and aids in orthognathic surgical planning. These tools are essential for digital treatment planning, providing orthodontists and surgeons with the ability to simulate surgeries, design splints, and visualize the outcomes.

Recent advancements in virtual surgical planning (VSP) and digital surgical splint design have significantly enhanced the precision and outcomes of facial surgeries, particularly in orthognathic procedures and crown lengthening. Meshmixer (Autodesk Inc., California) plays a role in these advancements by enabling the editing and repair of 3D meshes for diagnostic and presurgical modeling. It supports creating virtual models used in VSP to assist in precise jaw adjustments using cephalometric X-rays and photos [28]. Maestro 3D (AGE Solutions, Italy) has also been integrated into VSP workflows, particularly with Dolphin Imaging software (Patterson Dental Holdings, USA). A study confirmed that this combination enhances the clinical reliability of VSP, improving the precision of orthognathic surgical planning by enabling detailed 3D modeling of the facial structures and jaw alignment before surgery [36]. Materialise Mimics (Materialise, Belgium), a more specialized 3D modeling software, further advances VSP by converting medical images into accurate 3D models for cranio-maxillofacial surgery and dental implant planning. It supports the creation of custom 3D-printed guides for surgery, improving both the efficiency and accuracy of procedures. By predicting post-surgery soft tissue changes, it ensures that surgeons can better plan for optimal aesthetic and functional outcomes. The workflow facilitated by Materialise includes uploading CT scans, collaborating on treatment planning, and utilizing 3D-printed guides during surgery [37].

Together, these software demonstrate the possibilities of digital technologies in revolutionizing surgical planning, from improved diagnostics and modeling to custom surgical guides, ultimately leading to more successful patient outcomes in facial surgeries and dental procedures.

Treatment planning

At present, orthodontic treatment planning involves the use of advanced software tools that enable orthodontists to analyze, design, and plan treatments more accurately and efficiently. These software tools integrate advanced imaging, 3D modeling, and AI to enhance the planning and execution of orthodontic treatments. They support various treatment approaches, from clear aligners to traditional braces.

The SureSmile (Dentsply Sirona, USA) software leverages 3D imaging and robotics to streamline orthodontic workflows. It customizes appliance designs, including arch wires, with robotic precision, improving both treatment accuracy and efficiency. This technology enables orthodontists to plan treatments more effectively and execute wire designs with greater precision, reducing the overall treatment time [31]. Digital Smile Design (DSD, Brazil) revolutionizes smile aesthetics by using photographs to create personalized smile designs. DSD improves the aesthetic analysis of a patient's smile and enhances communication between dental professionals and patients. Providing a digital mock-up of the final smile helps patients visualize the potential outcome, leading to more predictable treatment results [38]. However, DSD requires precise documentation, and specialized training, and can be costly. Common software tools used in DSD include Photoshop, Exocad, and Smile Designer Pro, all of which allow for detailed adjustments in smile design based on facial and dental features [39]. The Dentone (DIORCO, Korea) software is designed to improve diagnosis, treatment planning, and patient management. It integrates advanced features like 3D imaging, cephalometric analysis, and digital treatment simulations, enhancing precision and efficiency in orthodontic practices. It streamlines workflows by providing detailed visualizations of treatment progress, automating patient management tasks, and supporting digital diagnostics through integration with CBCT and cephalometric data [18]. Both SureSmile and DSD offer significant advantages in orthodontics and cosmetic dentistry. The SureSmile software focuses on optimizing orthodontic treatment with precise, technology-driven planning, whereas DSD enhances the patient experience by improving communication and enabling highly personalized aesthetic designs.

Indirect bonding

The indirect bonding planning software tools optimize the indirect bonding process by offering accurate bracket placement, reducing manual steps, and enabling the use of digital models and 3D printing. This leads to enhanced precision and efficiency, reducing chair time and improving treatment outcomes.

Ortho Analyzer (3Shape, Denmark) plays a key role in the digital workflow for IDB by creating digital models from intraoral scans. It enables virtual bracket placement and setup design, generating STL files for 3D printing transfer trays that ensure accurate bracket positioning. This software streamlines the entire IDB process, helping orthodontists avoid errors and achieve optimal bracket placement, which is critical for the success of the treatment [40]. SureSmile (Dentsply Sirona, USA) also supports indirect bonding (IDB), along with custom archwire fabrication by facilitating the creation of 3D-printed IDB trays, especially useful during the finishing stages of orthodontic treatment to ensure more precise results [41]. Maestro 3D (AGE Solutions, Italy) enhances IDB by automating bracket placement and generating 3D-printed transfer trays, offering another level of precision. This automation reduces human error and accelerates the treatment process, making the workflow smoother and more efficient for orthodontists [25].

Together, these software tools provide a powerful suite of technologies that improve the accuracy, speed, and outcomes of orthodontic treatments, particularly in the critical stages of bracket placement and archwire customization.

Metal 3D printing

Metal 3D printing software solutions allow clinicians to design, simulate, and fabricate customized orthodontic appliances using 3D metal printing technology. This technology offers advantages such as increased strength, customization, and a precise fit, leading to better patient outcomes [42].

Exocad DentalCAD (Align Technology, USA) is known for its seamless integration with 3D printers, which streamlines the workflow for designing and printing orthodontic devices. Its direct design transfer and file management capabilities via ExoPrint significantly boost productivity and efficiency in orthodontic practices. It is more efficient than general-purpose software like Blender and Meshmixer due to its specific orthodontic focus and has better workflow integration than Blue Sky Plan, which is broader in scope [43]. Meshmixer (Autodesk Inc, California) is more versatile in model manipulation than Exocad and Blue Sky Plan, which are more specialized and offer greater flexibility at no cost, unlike 3Shape and Materialise, which are more focused on precision and workflow [43]. Blue Sky Plan (Bluesky Bio, USA) offers comprehensive 3D dental planning for orthodontics and surgery. It integrates DICOM and 3D models for precise arch alignment, guided surgery, and the design of 3D-printed surgical guides and aligners, enhancing the accuracy and outcome of dental procedures. It offers comprehensive planning features not available in Meshmixer and Blender [28]. 3Shape (3Shape, Denmark) is widely used for its precision in creating dental models, surgical guides, and orthodontic devices. Its compatibility with 3D printers like Formlabs and Stratasys ensures high-quality custom aligners and appliances, improving efficiency and accuracy in dental treatments. Provides better integration and precision than Meshmixer and Blender, which are more generic [23]. Blender (The Blender Foundation, Netherlands) excels in occlusal splints and model alignment. Its versatility and integration with other tools like Autodesk Netfabb and Meshmixer allow for detailed refinement of STL files, making it a powerful tool for custom dental device creation. Less precise but more flexible than 3Shape and Materialise, making it suitable for a wide range of applications [28]. Materialise Magic Dental Module (Materialise, Belgium) optimizes 3D printing workflows in dental labs, offering cost savings, scalability, and efficient digital processes. It supports the entire workflow from design import to printing, and is particularly useful for creating crowns, bridges, periodontal scaffolds, and orthodontic devices, enhancing precision and treatment quality. They are more focused on optimizing lab workflows and scalability than Meshmixer and Blender. They provide a more comprehensive and precise workflow than Exocad and Blue Sky Plan for lab environments [37].

Face scan and 3D planning

Face scan software in orthodontics employs 3D imaging to evaluate facial features, dental alignment, and aesthetics. By integrating facial scans with intraoral and CBCT data, it improves diagnosis, treatment planning, and progress monitoring. This combination facilitates personalized, precise, and aesthetically aligned orthodontic care.

Planmeca Romexis (Planmeca Oy, Finland) offers superior diagnostics by integrating 3D facial and CBCT data, enhancing treatment accuracy. It provides automatic tracing, treatment overlays, and precise surgical simulation, streamlining cephalometric analysis and treatment planning [44]. DentSim (Image Navigation, Israel) is ideal for training with realistic orthodontic simulations and facial imaging integration [45]. CEREC (Dentsply Sirona, USA) enables real-time 3D visualization for both cosmetic and functional treatment planning [23]. Exocad (Align Technology, USA) enhances efficiency in digital dentistry by enabling seamless integration of facial and intraoral scans using advanced algorithms. It supports more accurate 3D visualizations, leading to better esthetic outcomes and improved communication with patients and dental professionals [23]. OrthoCAD (Align Technology, USA) integrates digital models, 3D facial scans, and CBCT imaging for precise treatment simulations and improved patient communication. Digital impressions are easily stored and transferred, simplifying appliance fabrication with faster, more predictable outcomes [46]. KODAK 3D Imaging (Carestream Dental, USA) is perfect for complex cases, integrating facial scans and CBCT data for precise treatment planning. 3D planning enhances anatomical understanding by allowing surgeons to practice procedures virtually before real surgery. It also serves as an effective tool for training, case presentation, and clear communication with colleagues and patients [47]. Meshmixer (Autodesk Inc., California) enables 3D mesh editing and digital diagnostic waxing using tooth libraries. It allows the transfer of 2D smile design into 3D for accurate treatment planning [23]. Meshlab (Visual Computing Lab, Italy) processes 3D data for alignment and modifications but does not support creating virtual wax-ups, limiting its application in restorative design [23]. Titan (Titan Dental Design, USA) uses AI-driven technology for facial scanning and orthodontic treatment planning. It offers mobile-based face scanning and supports automated dental scan segmentation, force field visualization, and aligner production management [13]. Blender (The Blender Foundation, Netherlands) has dental-specific features, including an articulator tool that integrates 2D facial scans and intraoral digital scans. It uses the iterative point algorithm to merge these and mount diagnostic casts into a virtual articulator for facially driven diagnostic waxing and treatment planning [23].

Limitations of the current software in orthodontics

Recent advancements in orthodontic software tools incorporating AI have significantly improved diagnosis, treatment planning, and patient monitoring. However, there are still some limitations to consider: 1) The biological variations in patient anatomy, compliance, and treatment responses may affect the software's accuracy [48]. 2) The healthcare systems are legally bound to patient data security and privacy. The software needs to be compliant with regional legislation and regulatory norms like HIPAA and DISHA [48,49]. 3) The economic constraints often limit access to advanced orthodontic software in smaller clinical practices [50]. 4) The newly developed software may pose a steep learning curve could invariably reduce usage [28]. 5) Software with complex interfaces may have difficulty integrating with other systems. 6) Software systems designed to monitor treatment progress have limitations on relapse detection and ensuring long-term stability of treatment outcomes [48]. 7) Extensive reliance on software may bias clinical decision-making and raise ethical concerns [48].

## Conclusions

Advances in dental software and digital technologies have improved orthodontic planning, diagnosis, and patient management. AI-driven tools automate cephalometric analysis and digital aligner fabrication, which reduces errors. Smile design and 3D printing streamline appliance creation, reducing delays. Virtual surgical planning enhances the precision of orthognathic surgery. Continuous AI integration and research are essential for further increasing the accuracy and validity of software-generated treatment outcomes.

## References

[1] Monill-González A, Rovira-Calatayud L, d'Oliveira NG, Ustrell-Torrent JM (2021). Artificial intelligence in orthodontics: where are we now? A scoping review. Orthod Craniofac Res.

[2] Subramanian AK, Chen Y, Almalki A, Sivamurthy G, Kafle D (2022). Cephalometric analysis in orthodontics using artificial intelligence-a comprehensive review. Biomed Res Int.

[3] Mahto RK, Kafle D, Giri A, Luintel S, Karki A (2022). Evaluation of fully automated cephalometric measurements obtained from web-based artificial intelligence driven platform. BMC Oral Health.

[4] Power G, Breckon J, Sherriff M, McDonald F (2005). Dolphin Imaging Software: an analysis of the accuracy of cephalometric digitization and orthognathic prediction. Int J Oral Maxillofac Surg.

[5] Kumar M, Kumari S, Chandna A, Konark Konark, Singh A, Kumar H, Punita Punita (2020). Comparative evaluation of CephNinja for android and nemoceph for computer for cephalometric analysis: a study to evaluate the diagnostic performance of CephNinja for cephalometric analysis. J Int Soc Prev Community Dent.

[6] Gregston M, Kula T, Hardman P, Glaros A, Kula K (2004). A comparison of conventional and digital radiographic methods and cephalometric analysis software: I. hard tissue. Semin Orthod.

[7] Mohan A, Sivakumar A, Nalabothu P (2021). Evaluation of accuracy and reliability of OneCeph digital cephalometric analysis in comparison with manual cephalometric analysis-a cross-sectional study. BDJ Open.

[8] Naoumova J, Lindman R (2009). A comparison of manual traced images and corresponding scanned radiographs digitally traced. Eur J Orthod.

[9] Ravera S, Castroflorio T, Garino F, Daher S, Cugliari G, Deregibus A (2016). Maxillary molar distalization with aligners in adult patients: a multicenter retrospective study. Prog Orthod.

[10] İzgi E, Pekiner FN (2019). Comparative evaluation of conventional and OnyxCeph™ dental software measurements on cephalometric radiography. Turk J Orthod.

[11] Bazina M, Cevidanes L, Ruellas A (2018). Precision and reliability of Dolphin 3-dimensional voxel-based superimposition. Am J Orthod Dentofacial Orthop.

[12] Logan S, Riedy CA, Hargett K, Katebi N (2024). Orthodontists' use of remote monitoring platforms pre-, amid, and post-COVID-19: a survey study. BMC Oral Health.

[13] Kazimierczak N, Kazimierczak W, Serafin Z, Nowicki P, Nożewski J, Janiszewska-Olszowska J (2024). AI in orthodontics: revolutionizing diagnostics and treatment planning-a comprehensive review. J Clin Med.

[14] Caruso S, Caruso S, Pellegrino M, Skafi R, Nota A, Tecco S (2021). A knowledge-based algorithm for automatic monitoring of orthodontic treatment: the dental monitoring system. two cases. Sensors (Basel).

[15] Yacout YM, Eid FY, Tageldin MA, Kassem HE (2024). Evaluation of the accuracy of automated tooth segmentation of intraoral scans using artificial intelligence-based software packages. Am J Orthod Dentofacial Orthop.

[16] Felter M, Lenza MM, Lenza MG, Shibazaki WM, Silva RF (2018). Comparative study of the usability of two software programs for visualization and analysis of digital orthodontic models. J Dent Res Dent Clin Dent Prospects.

[17] Ghafoor H (2018). Reverse engineering in orthodontics. Turk J Orthod.

[18] Kharbanda R, Sharma S, Agrawal N (2021). Softwares in orthodontics- a review. Int J Sci Res.

[19] Chenin DL, Chenin DA, Chenin ST, Choi J (2009). Dynamic cone-beam computed tomography in orthodontic treatment. J Clin Orthod.

[20] Koretsi V, Tingelhoff L, Proff P, Kirschneck C (2018). Intra-observer reliability and agreement of manual and digital orthodontic model analysis. Eur J Orthod.

[21] Savić MA, Savić M, Arbutina A, Umićević-Davidović M, Mirjanić V (2017). A system for measurements of 3D scanned orthodontic study models. Contemp Mater.

[22] Porto BG, Porto TS, Silva MB (2014). Comparison of linear measurements and analyses taken from plaster models and three-dimensional images. J Contemp Dent Pract.

[23] Piedra-Cascón W, Fountain J, Att W, Revilla-León M (2021). 2D and 3D patient's representation of simulated restorative esthetic outcomes using different computer-aided design software programs. J Esthet Restor Dent.

[24] Dhingra A, Palomo JM, Stefanovic N, Eliliwi M, Elshebiny T (2022). Comparing 3D tooth movement when implementing the same virtual setup on different software packages. J Clin Med.

[25] Raju R, Tr PA (2024). Accuracy of tooth segmentation in the digital kesling setup of two different software programs: a retrospective study. Cureus.

[26] Panayi NC (2023). Directly printed aligner: aligning with the future. Turk J Orthod.

[27] Tabancis NN, Krey KF, Stahl F, Behnke V, Ratzmann A (2023). Orthodontic treatment and biological limits: a retrospective clinical trial. Head Face Med.

[28] Federici Canova F, Oliva G, Beretta M, Dalessandri D (2021). Digital (r)evolution: open-source softwares for orthodontics. Appl. Sci.

[29] Narongdej P, Hassanpour M, Alterman N, Rawlins-Buchanan F, Barjasteh E (2024). Advancements in clear aligner fabrication: a comprehensive review of direct-3D printing technologies. Polymers (Basel).

[30] (2022). An innovation revolution. Br Dent J.

[31] Sachdeva RC (2001). SureSmile technology in a patient--centered orthodontic practice. J Clin Orthod.

[32] Sodagar A, Rafatjoo R, Gholami Borujeni D, Noroozi H, Sarkhosh A (2010). Software design for smile analysis. J Dent (Tehran).

[33] Finelle G (2017). Digital smile design in interdisciplinary and orthodontic dental treatment planning. J Dentofac Anom Orthod.

[34] Nasri S, Gassara Y, Kallala R (2024). Digital smile design software: an overview. Sch J Med Case Rep.

[35] Girish V, Vijayalakshmi A (2004). Affordable image analysis using NIH Image/ImageJ. Indian J Cancer.

[36] Elshebiny T, Morcos S, Mohammad A, Quereshy F, Valiathan M (2019). Accuracy of three-dimensional soft tissue prediction in orthognathic cases using dolphin three-dimensional software. J Craniofac Surg.

[37] Marchetti C, Bianchi A, Muyldermans L, Di Martino M, Lancellotti L, Sarti A (2011). Validation of new soft tissue software in orthognathic surgery planning. Int J Oral Maxillofac Surg.

[38] Thomas PA, Krishnamoorthi D, Mohan J, Raju R, Rajajayam S, Venkatesan S (2022). Digital Smile Design. J Pharm Bioallied Sci.

[39] Stanley M, Paz AG, Miguel I, Coachman C (2018). Fully digital workflow, integrating dental scan, smile design and CAD-CAM: case report. BMC Oral Health.

[40] Bachour PC, Klabunde R, Grünheid T (2022). Transfer accuracy of 3D-printed trays for indirect bonding of orthodontic brackets. Angle Orthod.

[41] Gündoğ H, Arman Özçırpıcı A, Pamukçu H (2023). Transfer accuracy of three indirect bonding trays: an in vitro study with 3D scanned models. Turk J Orthod.

[42] Dawood A, Marti Marti B, Sauret-Jackson V, Darwood A (2015). 3D printing in dentistry. Br Dent J.

[43] Abad-Coronel C, Pazán DP, Hidalgo L, Larriva Loyola J (2023). Comparative analysis between 3D-printed models designed with generic and dental-specific software. Dent J (Basel).

[44] Chinchilla-Torres P, Rodríguez-Pacheco MJ, Chinchilla-Herrera R (2024). Comparative analysis of the discrepancy in conventional cephalometric tracing and digital cephalometric tracing with planmeca romexis® software. Odovtos - Int J Dent Sc.

[45] Dzhendov D, Gergana G (2022). The application of simulators in dental medicine students’ training. Scripta Scientifica Medica.

[46] Taneva E, Kusnoto B, Evans CA (2015). 3D Scanning, Imaging, and Printing in Orthodontics. Issues in contemporary orthodontics.

[47] Rubio-Palau J, Prieto-Gundin A, Cazalla AA, Serrano MB, Fructuoso GG, Ferrandis FP, Baró AR (2016). Three-dimensional planning in craniomaxillofacial surgery. Ann Maxillofac Surg.

[48] Strunga M, Urban R, Surovková J, Thurzo A (2023). Artificial intelligence systems assisting in the assessment of the course and retention of orthodontic treatment. Healthcare (Basel).

[49] Mrinmoy R, Aggarwal L (2023). A strategic roadmap to the successful implementation of digital health records in India.

[50] Camardella LT, Rothier EK, Vilella OV, Ongkosuwito EM, Breuning KH (2016). Virtual setup: application in orthodontic practice. J Orofac Orthop.

